# Beyond a Simple Switch: Decoding the Multifactorial Phenotypic Plasticity of Vascular Smooth Muscle Cells

**DOI:** 10.3390/cells14151171

**Published:** 2025-07-30

**Authors:** Francisca Muñoz, Claire M. Holden, Alejandra San Martin

**Affiliations:** 1 Institute of Biomedical Sciences, Faculty of Medicine and Faculty of Life Sciences, Universidad Andres Bello, Santiago 7591538, Chile; fmuoz@uc.cl; 2 Department of Medicine, Division of Cardiology, Emory University, Atlanta, GA 30322, USA; claire.holden@emory.edu

**Keywords:** vascular smooth muscle cell, vasculature, phenotypic modulation, cardiovascular diseases, atherosclerosis, restenosis, epigenetics

## Abstract

Vascular smooth muscle cells (VSMCs) are central to the maintenance of vascular homeostasis and the progression of cardiovascular diseases (CVDs), owing to their remarkable phenotypic plasticity. This editorial introduces a Special Issue of *Cells* that compiles recent advances in our understanding of the molecular, epigenetic, metabolic, and mechanical mechanisms that govern VSMC behavior. Highlighted contributions explore the roles of RNA modifications, chromatin remodeling, lipid metabolism, and mechanotransduction in VSMC phenotypic switching, revealing new therapeutic targets and diagnostic opportunities. Together, these studies emphasize the multifactorial regulation of VSMC plasticity and its dual role in vascular repair and disease pathogenesis.

Cardiovascular diseases (CVDs) continue to be a leading cause of mortality worldwide. Central to vascular health and disease are vascular smooth muscle cells (VSMCs). These cells, vital for maintaining vessel integrity and responsiveness, exhibit remarkable phenotypic plasticity. While this intrinsic adaptability is crucial for vascular repair, it is also a key factor in various cardiovascular pathologies. The full extent of VSMC contributions to vascular biology and disease is, in fact, continually evolving. These ongoing discoveries reveal a complexity that encompasses diverse sub-phenotypes, intricate regulatory mechanisms, and untapped therapeutic potential, extending far beyond earlier understandings.

From atherosclerosis and aneurysm formation to restenosis and vascular remodeling, the transition of VSMCs from a quiescent, contractile state to a synthetic, inflammatory, osteogenic, or macrophage-like phenotype is a recognized factor in vascular disease progression [1]. This Special Issue of *Cells* consists of 12 original research articles and reviews that explore the dynamic contributions of VSMCs to cardiovascular pathology, highlighting novel molecular mechanisms, regulatory pathways, and potential therapeutic targets.

Recent advances emphasize the importance of post-transcriptional regulation in controlling VSMC phenotypic plasticity. Among emerging post-transcriptional regulators, the m^6^A reader protein YTHDF1 (YTH N6-methyladenosine RNA Binding Protein 1) has garnered increasing attention. In this Special Issue, Tian et al. demonstrate that YTHDF1 plays a protective role in VSMCs by preserving their contractile phenotype and attenuating neointimal hyperplasia following vascular injury (Contribution 1). Mechanistically, YTHDF1 expression declines early during PDGF-BB–induced phenotypic modulation, preceding the downregulation of contractile markers. Restoration of YTHDF1 expression, both in vitro and in vivo, reverses this phenotypic shift and inhibits pathological remodeling, identifying YTHDF1 as a novel regulator of VSMC plasticity and a potential therapeutic target in vascular disease. While this phenotypic modulation contributes to vascular pathogenesis, it also represents an adaptive response vital for tissue repair following injury. Similarly, RNA editing mediated by ADAR1 is shown to be essential for VSMC viability; its deletion in murine models leads to severe vascular dysfunction and lethality (Contribution 2).

Within this regulatory framework, epigenetic mechanisms exert profound control over VSMC phenotypic transitions [2]. The SWI/SNF chromatin remodeling complex, for instance, modulates gene expression programs that enable VSMCs to dynamically respond to vascular injury and stress (Contribution 3). Expanding on epigenetic contributions to VSMC phenotypic plasticity, Yan et al. examined the vascular effects of (+)-JQ1, a widely used BET bromodomain inhibitor. Surprisingly, their findings reveal that (+)-JQ1 reduces VSMC contractility via mechanisms independent of BET inhibition (Contribution 4). Specifically, (+)-JQ1 inhibits the phosphorylation of myosin light chain 20 (LC20), blocking actin–myosin interaction and suppressing the contractile response. These effects occur through both endothelium-dependent (via eNOS activation) and endothelium-independent (via reduced calcium influx) pathways. Importantly, similar outcomes were observed with the inactive enantiomer (−)-JQ1, highlighting potential off-target actions of JQ1 and emphasizing the need for caution when interpreting vascular phenotypes in studies using this compound.

These epitranscriptomic and epigenetic mechanisms coordinate with extracellular signaling pathways to maintain the contractile VSMC phenotype. Cullen et al. showed that adiponectin, an adipokine, activates the AKT pathway, promoting contractile gene expression and inhibiting PDGF-BB–induced proliferation, thus reinforcing the contractile identity of human VSMCs (Contribution 5). In addition, the calcium/calmodulin-dependent phosphatase calcineurin facilitates the nuclear translocation of NFAT, regulating genes involved in VSMC proliferation, migration, and differentiation, and modulating ion channels and cellular receptors that influence vascular tone (Contribution 6). Similarly, Rager et al. (Contribution 7) reported that natriuretic peptide receptors (NPR-A and NPR-B), activated by ANP and BNP, increase intracellular cGMP levels in VSMCs, reducing contraction, inhibiting proliferation and migration, and attenuating plaque formation and vascular stenosis. Together, these epitranscriptomic, epigenetic, and extracellular signaling pathways converge to dynamically regulate VSMC phenotype in both vascular health and disease.

Lipid metabolism has emerged as a key determinant of VSMC phenotypic plasticity under pathological conditions. Work presented in this Special Issue also implicated the enzyme fatty acid synthase (FASN), which catalyzes de novo fatty acid synthesis, in the pathological reprogramming of these cells. FASN is upregulated in VSMCs and promotes their transdifferentiation into a foam cell-like phenotype in cholesterol-rich environments, enhancing atherosclerotic plaque formation. This shift suggests that lipid-derived environmental cues, like those induced by the presence of cholesterol, may activate specific metabolic pathways that induce intracellular lipid accumulation and drive VSMC conversion toward a pro-atherogenic state (Contribution 8).

Beyond classical molecular pathways, mechanotransduction represents another crucial mechanism regulating VSMC phenotypic plasticity. The Lipoma-Preferred Partner (LPP) was identified as an SMC-specific mechanotransducer that senses biomechanical changes and translates them into cellular responses (Contribution 9). LPP remodels the cytoskeletal architecture in response to mechanical tension, preserving a contractile phenotype and vascular myogenic function. Conversely, loss of LPP leads to a synthetic, migratory, and proliferative state, impairing the arterial myogenic response and contributing to pathological vascular remodeling. In parallel, the cadherin FAT1 promotes VSMC migration by reorganizing the cytoskeleton via the Ena/VASP pathway. FAT1 expression increases in response to mechanical and hormonal stimuli, such as vascular injury or angiotensin II, through the NOX1–ROS–ERK1/2 axis. Inhibition of FAT1 significantly reduces VSMC migration, highlighting its potential as a therapeutic target in pathological vascular remodeling (Contribution 10).

As discussed, while essential for tissue adaptation, the phenotypic plasticity of VSMCs can also lead to pathological outcomes. A clear example is vascular calcification, where VSMCs acquire an osteogenic phenotype [3]. Under stress conditions—such as exposure to inorganic phosphate and biomechanical stimuli—these cells upregulate the expression of bone morphogenetic proteins BMP-2 and BMP-4, along with their receptors, activating a transcriptional response induced by the activation of RUNX2. This transdifferentiation program drives a shift toward an osteoblast-like phenotype, promoting vascular wall calcification and increasing arterial stiffness—key mechanisms in the progression of diseases like atherosclerosis. These effects have been characterized using dynamic in vitro models by Cabiati et al. (Contribution 11).

Regarding advances in experimental methodology, Rager et al. validated the U2 gene as a reliable reference marker for the normalization of gene expression in VSMCs, both in culture and in tissue (Contribution 12). The use of U2 as a stable reference gene enables more consistent comparisons between in vitro and ex vivo gene expression, improving the rigor of VSMC research.

In conclusion, the evidence presented in this Special Issue of *Cells* unequivocally demonstrates that VSMC phenotypic plasticity is regulated by a myriad of factors, primarily a complex interplay of epigenetic, epitranscriptomic, metabolic, and mechanical pathways, as well as extracellular signals. While this capacity for phenotypic change allows VSMCs to respond dynamically to various physiological stimuli, its dysregulation under pathological conditions profoundly contributes to the development and progression of cardiovascular diseases. Therefore, a detailed understanding of these intricate processes is crucial for the design of novel therapeutic and diagnostic strategies aimed at combating the global burden of cardiovascular disease.

Collectively, these studies deepen our understanding of the mechanisms governing VSMC behavior in health and disease (Figure 1). They highlight VSMC’s inherent cellular heterogeneity and functional adaptability, as well as therapeutic opportunities that arise from targeting their maladaptive responses. As our insights into VSMC biology continue to grow, so does our potential to develop more targeted and effective strategies for the treatment of vascular diseases.

## Figures and Tables

**Figure 1:**
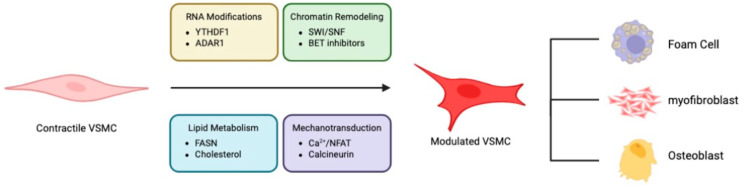
Multiple mechanisms drive the formation of alternative phenotypes from fully differentiated VSMCs.
